# Immunotherapy in Prostate Cancer

**DOI:** 10.3390/cancers12071752

**Published:** 2020-07-01

**Authors:** Emily K. Fay, Julie N. Graff

**Affiliations:** 1 Knight Cancer Institute, Oregon Health & Science University, Portland, OR 97239, USA; fayem@ohsu.edu; 2 VA Portland Health Care System, Portland, OR 97239, USA

**Keywords:** prostate cancer, checkpoint inhibitor, vaccine, immunotherapy

## Abstract

Immunotherapy encompasses a wide range of therapies to engage the immune system to target malignancies. In recent years, immunotherapy has made a major impact on treatment of metastatic cancer and has altered standard of care for many tumor types. However, predicting and understanding responses across tumor types has been challenging. While some metastatic cancers have shown dramatic responses to immunotherapy, such as melanoma, lung cancer, and renal cell carcinoma, prostate cancer has generally failed to show a significant response. However, small series of prostate cancer patients have shown impressive responses to cellular and immunotherapy. This review summarizes the current data for immunotherapy’s use in prostate cancer, as well as how currently available data might help predict patient responses to immunotherapy. Specifically, we will review vaccine-based therapies, immune checkpoint inhibitors, and future directions that are actively being explored.

## 1. Immunotherapy in Prostate Cancer

Immunotherapy encompasses a wide range of therapies to engage the immune system to target malignancies. In recent years, immunotherapy has made a major impact on the treatment of metastatic cancer and has altered the standard of care for many tumor types. However, predicting and understanding responses across tumor types has been challenging. While some metastatic cancers have shown dramatic responses to immunotherapy, such as melanoma, lung cancer, and renal cell carcinoma, prostate cancer has generally failed to show a significant response. However, a small series of prostate cancer patients have shown impressive responses to cellular and immunotherapy, suggesting that it is worthy of further study. This review summarizes the current data for immunotherapy’s use in prostate cancer, as well as how currently available data might help predict patient responses to immunotherapy. Specifically, we will review vaccine-based therapies, immune checkpoint inhibitors, and future directions that are actively being explored.

## 2. Prostate Tumor Microenvironment

Prostate cancer is often described as a “cold” tumor, with an immunosuppressive microenvironment. Tumor-infiltrating lymphocytes (TILs) may contribute to prostate cancer progression by inhibiting the activity of T-effector cells. Prostate cancer biopsy specimens have been shown to have TILs that are skewed towards T-regulatory (Treg) and T helper 17 (Th17) phenotypes, which suppress autoreactive T cells and antitumor immune responses [1].

There is interest in designing therapies that could enhance immune infiltration by antigen-presenting cells (APCs) and effector T cells. Dendritic cells (DCs) are antigen-presenting cells that are important in CD8+ T cell activation and subsequent tumor killing. Several studies have associated DC tumor infiltration with improved prognosis [2]. Androgen deprivation therapy (ADT) has been shown to temporarily mitigate T cell tolerance and induce T cell priming to prostatic antigens [3,4]. This suggests that the combination of ADT with immunotherapy may be synergistic.

Specific host factors may also play a role in prostate cancer tumorigenesis and response to immunotherapy. Multiple studies show that the composition of the gut microbiota may impact responses to chemotherapy and immunotherapy and that antibiotics may blunt response to treatment [5,6,7]. A recent study showed high levels of CD8+ TILs in patients treated with checkpoint inhibitors (CPIs) who had gut microbiomes predominated by the Ruminococcaeae family, while patients with a Bacteroidales-predominant gut microbiome had more Tregs and reduced cytokine activity [8].

Currently, the optimal combination and sequencing of immunotherapies are poorly understood. Immunotherapy has been explored in prostate cancer as single-agent therapy and in combination with other immunotherapies, as we will review below.

## 3. Vaccine-Based Therapies

Prostate cancer cells express several immunogenic antigens that are specific to the prostate, including prostatic acid phosphatase (PAP) and prostate-specific antigen (PSA), both of which have been explored as targets for antigen-based vaccines [9]. Sipuleucel-T is an autologous cellular immunotherapy administered by vaccine, created by collecting a patient’s dendritic cells by leukapheresis, incubating them with a prostate cancer-associated antigen at a central processing facility, and ultimately re-administering the engineered product to the patient through reinfusion [10]. The antigen, PA2024, is a recombinant fusion protein involving prostatic acid phosphatase (PAP), which is expressed in the vast majority of prostate adenocarcinomas and is specific to prostate tissue, and granulocyte macrophage colony-stimulating factor (GM-CSF), a cytokine involved in immune cell maturation and activation. The receptor for GM-CSF is broadly expressed on antigen-presenting cells (APCs). When exposed to PAP in vitro, APCs were shown to induce cytotoxic T-lymphocytes to recognize and kill prostate tumor cells [11,12]. When PAP was conjugated with GM-CSF in animal studies, greater antigen-specific immune responses were seen [12,13]. The recombinant protein is incubated with the patient’s APCs ex vivo to allow APCs to display this antigen on their surface, and the engineered APCs are then reinfused in the patient.

Sipuleucel-T has been evaluated in three Phase III clinical trials (NCT00065442, NCT00005947, and NCT01133704), the largest of which, and the basis for sipuleucel-T’s Federal Drug Administration approval in 2010, is the multicenter Immunotherapy for Prostate Adenocarcinoma Treatment (IMPACT trial). Patients in the IMPACT trial were allowed to have mildly symptomatic disease but could not have visceral metastases, pathologic bone fractures, spinal cord compression, or treatment in the previous months with systemic glucocorticoids, radiation, surgery, or systemic therapy for prostate cancer [14]. Patients were randomized 2:1 to receive sipuleucel-T or placebo, given at weeks 0, 2, and 4. Overall survival (OS) was the primary study endpoint. Of patients assigned to placebo, they were offered sipuleucel-T at disease progression as salvage.

Median survival in the sipuleucel-T-treated patients was 25.8 months, compared to 21.7 months in placebo-treated patients. There was no difference observed in time to progression of the disease. The most common adverse events seen in the sipuleucel-T arm were fevers, chills, headache, influenza-like illness, myalgias, hypertension, hyperhidrosis, and groin pain [14].

The IMPACT trial has been subject to several criticisms. Importantly, in IMPACT’s design, patients on the sipuleucel-T arm were given the next line of treatment at disease progression, frequently docetaxel, while patients on the placebo arm were given sipuleucel-T at disease progression. This resulted in a delay in receiving a therapy known to be effective for prostate cancer [15]. Importantly, in addition to no change in progression-free survival (PFS), no significant impacts on PSA, tumor burden, symptoms, or pain have been observed from sipuleucel-T. Without a meaningful impact on surrogate endpoints, it is hard to understand and explain sipuleucel-T’s observed improvement in overall survival. In addition, there is no marker in the blood, or otherwise, that can predictably tell clinicians and patients if it is working.

Despite the lack of clear anticancer activity with sipuleucel-T, there have been case reports of dramatic responses to this vaccine. One case of a lasting response to sipuleucel-T was noted in the Phase II study evaluating the efficacy of sipuleucel-T. One patient in this study experienced a decline in PSA from 221 ng/mL to undetectable by week 24, remaining undetectable at over 4 years from vaccine administration, and with a resolution of pelvic and retroperitoneal adenopathy [16]. It is not clear why this patient experienced such a dramatic antitumor response, while this was not re-demonstrated in the Phase III trials mentioned above. Interestingly, another case of a lasting response to sipuleucel-T was noted in a patient receiving concurrent enzalutamide. A patient with metastatic castrate-resistant prostate cancer (mCRPC) on enzalutamide and LHRH agonist therapy received sipuleucel-T after PSA rose from undetectable to 1.49 ng/mL. After 6 months, PSA unexpectedly declined, eventually becoming undetectable and remaining undetectable a year later, with no evidence of disease progression on scans [17]. The authors speculate that the response seen in this case may be related to androgen receptor blockers enhancing the effect of immunotherapy. Androgen ablation has been shown to mitigate T-cell tolerance to prostate cancer cells in a mouse model [3]. Additionally, the delayed response with PSA decline seen at 6 months does point to an immune-driven mechanism, as T-cell responses to vaccination generally take weeks to months to develop [18].

Another interesting observation is the effect of race on overall survival in patients treated with sipuleucel-T. The PROCEED registry is a multicenter, observational study of patients who received sipuleucel-T in one of three Phase III clinical trials. The registry evaluated 1902 men, 221 of whom were African American and 1649 of whom were Caucasian. Because PSA is known to differ significantly at baseline between these two racial groups, a PSA-matched cohort was created, which matched Caucasian patients to African Americans 2:1 with baseline PSA levels within +/− 10% [19].

Within the PSA-matched set, overall survival differed between African American men with a hazard ratio (HR) of 0.70 (95% CI: 0.57–0.86, *p* < 0.0001); within the all patient set, OS differed with an HR of 0.81 (95% CI: 0.68–0.97). Median OS for African Americans compared to Caucasians was 35.3 vs. 25.8 months in the PSA-matched set and 35.2 vs. 29.9 months in the all patient set. Adverse events (AEs) did not differ between the two groups. The reason for the observed difference in OS between races is not understood, though there is evidence that the biology of prostate cancer and its response to treatment differs between African Americans and Caucasians [20,21]. African Americans have long been underrepresented in clinical trials, and it remains important to continue narrowing this gap to help better understand these differences in treatment responses and to help inform treatment decisions based on unique patient factors.

## 4. PSA-TRICOM

Another vaccine therapy that has been evaluated for use in prostate cancer is the PSA-TRICOM vaccine (Prostvac). PSA-TRICOM uses a poxvirus which has been inserted with a recombinant plasmid containing a transgene for PSA, as well as plasmids for three human T-cell costimulatory molecules (TRICOM), including B7-1, ICAM-1, and LFA-3, to enhance T-cell response [22]. PSA-TRICOM is administered using a vaccinia-based vaccine as a priming vaccine, followed by monthly poxviral-based vaccines as booster vaccines. Unlike sipuleucel-T, PSA-TRICOM can be administered directly to patients without requiring the collection of a patient’s immune cells and ex vivo expansion.

An industry-sponsored Phase II study has shown the potential benefit of PSA-TRICOM in mCRPC. This trial randomized 125 patients in a 2:1 ratio to receive a priming dose of Prostvac followed by monthly boosters, or placebo, which was an empty vector vaccinia vaccine, followed by monthly empty vector fowlpox vaccine boosters [23]. The PSA-TRICOM arm was given GM-CSF on the day of vaccination and for 3 days after, while the placebo group received saline injections. Patients had to have minimally symptomatic mCRPC, confirmed by computerized tomographyor bone scan, and Gleason scores of ≤7, with evidence of PSA progression by Prostate-Specific Antigen Working Group criteria to be included [24]. The primary endpoint was PFS. The study did not reach its primary endpoint, but overall survival analysis showed a median survival of 25.1 months in the Prostvac arm compared to 16.6 months in the control arm [23].

A follow-up Phase III study further evaluated PSA-TRICOM vs. placebo in patients with mCRPC, as well as the role of GM-CSF as an adjuvant therapy, using OS as primary endpoint [25]. This study included three arms: Arm VG included patients receiving PSA-TRICOM + GM-CSF, Arm V included patients receiving PSA-TRICOM + placebo GM-CSF, and Arm P included patients receiving placebo vaccine + placebo GM-CSF. OS was 33.2 months in Arm VG, 34.4 in Arm V, and 34.3 in Arm P.

This study did not meet its primary endpoint or support the findings of improvement in survival, as seen in the Phase II study. Authors speculate that there may have been an imbalance in prognostic factors which negatively impacted survival in the Phase II control arm, as median OS was less than expected based on the Halabi prognostic nomogram for predicting OS (16.6 months vs. an expected 20.4 months) [26]. Importantly, during the treatment period for the Phase II study, the only life-prolonging treatment available for mCRPC was docetaxel. From the time the Phase III protocol was finalized until the last patient was randomly assigned, cabazitaxel, sipuleucel-T, abiraterone, enzalutamide, and radium-223 all became available as other options showing a survival benefit. PSA-TRICOM did not receive FDA-approval based on the findings of these trials.

There have also been efforts to combine prostate cancer vaccines with checkpoint inhibitors to enhance T-cell response, which has offered some of the earliest data for the use of checkpoint inhibition in prostate cancer. Checkpoint inhibitors (CPIs) are a type of immunotherapy that target immune checkpoints, barriers that can downregulate T-cell responses to promote self-tolerance. Cancer cells can also express immune checkpoints, leading to immune system evasion. Immune checkpoints that have been targeted in cancer treatment include cytotoxic T lymphocyte-associated antigen 4 (CTLA-4) and programmed death-1 (PD-1) [27,28]. As described below, the responses seen with CPI monotherapy have been disappointing thus far. This may be related to the absence of effector T cells within the tumor microenvironment. In a study evaluating neoadjuvant sipuleucel-T in patients who underwent radical prostatectomy for localized prostate cancer, patients who received sipuleucel-T were found to have a 3-fold increase in activated T cells in prostatectomy specimens compared to those who did not receive sipuleucel-T [29]. Thus, the combination of a cancer vaccine with CPIs, which further releases the “brake” set by immune checkpoints, is an attractive concept [30]. Preclinical models have shown enhanced antitumor activities from the combination of cancer vaccines with anti-PD-1/PD-L1 antibodies compared to PD-1 blockade or vaccine therapies given alone [31,32,33].

Both sipuleucel-T and PSA-TRICOM have been explored in combination with the CTLA-4 inhibitor ipilimumab. Preclinical data in mice have shown that CTLA-4 blockade can lead to enhanced T-cell mediated responses to vaccines [34]. A Phase I study of sipuleucel-T with low-dose ipilimumab (1 mg/kg) was performed in nine patients with mCRPC. Treatment was well-tolerated, with a Grade 1 rash in one patient as the only reported AE. Immunoglobulin levels to PA2024 and PAP were also shown to increase to higher levels for longer periods than what had been seen in previous Phase II and III studies with sipuleucel-T given alone [35,36].

A similar Phase I study of 30 patients with mCRPC looked at combining PSA-TRICOM with escalating doses of ipilimumab of 1, 3, 5, or 10 mg/kg, given with the first vaccine boost, followed by monthly ipilimumab infusions for up to 10 doses in total [37]. Authors noted a PSA decline in these patients similar to what was seen in patients receiving PSA-TRICOM only, with 58% of 24 chemotherapy-naïve patients experiencing a PSA decline. 70% of patients experienced a Grade 2 or greater immune-related adverse event, and 27% of patients had a Grade 3 or 4 adverse events. Current research is now focused on using prostate cancer vaccines in patients with low-risk localized prostate cancer. Sipuleucel-T is being investigated in a Phase III trial for patients with localized prostate cancer compared to patients undergoing active surveillance (NCT03686683), and PSA-TRICOM is being evaluated in a Phase II study, which will evaluate CD4+ and CD8+ cells within the prostate tumor and stroma pre- and post-treatment (NCT02326805). Other prostate cancer vaccines are also being explored in localized prostate cancer (NCT027768363, NCT03579654). It is not yet clear what role prostate cancer vaccines have, but they may be more beneficial when given early so that the immune system has time to mount a response. Vaccine administration could be more effective in the early stages of prostate cancer when the disease burden is low and prior to immune system evasion by the tumor [38].

## 5. CTLA-4 Inhibition

CTLA-4 blockade was first evaluated in prostate cancer in the transgenic adenocarcinoma of the mouse prostate (TRAMP) model, where the role of adjunctive CTLA-4 blockade in mice with minimal residual metastatic disease was studied. The TRAMP model mimics the pathogenesis of human prostate cancer, which, following primary resection, will frequently metastasize to regional lymph nodes and other organs following an interval of primary tumor growth [39]. In this study, mice underwent tumor resection, followed by adjuvant anti-CTLA-4 blockade or isotype-matched IgG on days 4, 7, and 10 after surgery. Mice without overt evidence of relapse were followed for at least 150 days after treatment. Macro- and micro-metastatic disease was quantified with immunohistochemistry. Of mice injected with CTLA-4 blockade, 44% (17/38) experienced relapse after primary tumor resection after at least 150 days of follow-up, whereas 97.4% (38/39) of mice receiving control antibody experienced relapse [40].

Radiation has been found to activate the immune response [41]. In a transgenic mouse model, radiation given prior to immunotherapy has been found to result in antitumor T-cell activation [42]. A Phase III study evaluated ipilimumab versus placebo after administration of radiotherapy in patients with mCRPC that had progressed after docetaxel [43]. In the CA184 043 study, patients received bone-directed radiotherapy of 8 Gy in one treatment fraction for up to 5 targets, which has been shown to be therapeutically equivalent to a fractionated regimen in terms of palliating pain [33,44]. Because radiotherapy was administered in both groups and was not controlled for, its impact on survival and other outcomes is unclear. After radiation, patients received ipilimumab 10 mg/kg or placebo every 3 weeks for up to four doses. Patients were followed with imaging every 12 weeks and PSAs every 6 weeks while on study therapy. Primary endpoint was overall survival, and secondary endpoints were PFS, pain response, and safety [43].

Overall survival in the ipilimumab group was 11.2 months, compared to 10.0 months in the placebo group, which was not statistically significant. Survival was less than expected in both treatment groups, possibly because so many patients had unfavorable baseline characteristics, including over 40% of patients being 80 or older, Gleason score > 7, and nearly a third of patients with visceral metastases [45].

Of note, the hazard ratio for death did decrease over time in the ipilimumab group, and at two years, overall survival was 26.2% in the ipilimumab group versus 15.0% in the placebo group. The authors did a post-hoc analysis looking at a subgroup of patients with baseline favorable prognostic features, including an alkaline phosphatase concentration of less than 1.5× upper limit of normal, a hemoglobin concentration of 110 g/L or higher, and no visceral metastases. In this subgroup, the authors noted that the median overall survival was 22.7 months in the ipilimumab group compared to 15.8 months in the placebo group. In all other patients, of whom all had at least one adverse prognostic feature, the median overall survival was 6.5 months in the ipilimumab group versus 7.3 months in the placebo group [43]. The authors postulated that immunotherapy may be more effective in patients with more favorable prognostic features, but that has never been studied in a prospective fashion. 

Ipilimumab was also evaluated in patients who had not previously received chemotherapy, with the hopes that this may offer greater benefit in patients who were less heavily pre-treated and had a lower burden of metastatic disease, particularly given the favorable subset described in the CA184 043 study. In the CA184 095 study, chemotherapy-naïve patients with asymptomatic or minimally symptomatic mCRPC without known visceral metastases were randomly assigned to ipilimumab 10 mg/kg versus placebo every 3 weeks for up to four doses, followed by double-blind maintenance treatment with ipiliumumab 10 mg/kg versus placebo every 12 weeks until intolerance or disease progression. Imaging was performed every 12 weeks until disease progression, with PSAs checked every 6 weeks. Pain intensity was also evaluated every 12 weeks using an analgesic use diary and a pain inventory form at each assessment point. The primary endpoint was OS; secondary endpoints were PFS, time to subsequent nonhormonal cytotoxic chemotherapy, and time to pain progression [46].

In total, 399 patients were treated with ipilimumab and 199 with placebo. OS was 28.7 months in the ipilimumab arm and 29.7 months in the placebo arm, with no significant difference observed between the groups. Interestingly, there was a significant difference seen in PFS between the two groups, 5.6 months in the ipilimumab group compared to 3.8 months in the placebo group. Treatment with ipilimumab also resulted in a significantly longer time to nonhormonal systemic therapy compared to placebo (HR 0.65; 95.87% CI, 0.52 to 0.83) as well as to docetaxel therapy (HR, 0.70; 95% CI, 0.55 to 0.88). PSA response rate was evaluated as an efficacy endpoint. There was a higher PSA response rate to ipilimumab (23%; 95% CI, 19% to 27%) compared to placebo (8%, 95% CI, 5% to 13%). Any-grade treatment-related adverse effects occurred in 82% of patients receiving ipilimumab and 49% receiving placebo, with 40% Grade 3 to 4 treatment-related AEs and 6%, respectively [46].

## 6. PD-1/PD-L1 Inhibition

PD-1 is an immune checkpoint receptor that is expressed on T-cells. When bound to programmed death ligand-1 (PD-L1), which is expressed on many peripheral tissues, it functions to suppress T-cell activity and promotes self-tolerance [28]. PD-L1 is also frequently expressed on tumor cells, resulting in their ability to evade the immune system. PD-L1 inhibition is being extensively studied across tumor types as a method of overcoming immune resistance. FDA-approved PD-1 inhibitors include nivolumab, pembrolizumab, cemiplimab, and FDA-approved PD-L1 inhibitors include atezolizumab, avelumab, and durvalumab.

In one of the first studies evaluating PD-1 inhibition with nivolumab across tumor types, objective responses were seen in patients with melanoma, renal cell carcinoma, and non-small cell lung cancer, but no objective responses (i.e., radiographic responses in soft-tissue) were observed in patients with prostate cancer, all of which were castrate-resistant [47]. In some tumor types, levels of PD-L1 expression correlate with response to treatment [48,49]. To date, clinical trials suggest that PD-L1 expression does not seem to correlate with response to PD-1 or PD-L1 inhibitors in prostate cancer. 

A Phase Ib basket trial evaluating pembrolizumab in patients with PD-L1 positive advanced cancers included a group of patients with PD-L1 positive mCRPC. Of 245 screened mCRPC patients, 35 (14%) men were found to be PD-L1 positive; 23 of these patients were enrolled and treated with pembrolizumab every 2 weeks for up to 2 years. Four patients experienced a partial response (PR), for an objective response rate (ORR) of 17% and a median duration of response of 13.5 months. Eight patients (35%) had stable disease, and nine (39%) had disease progression [50]. This study showed that although small in number, some patients derive durable benefit from PD-1 inhibition.

The largest study evaluating anti-PD-1 therapy in mCRPC thus far, Keynote 199, further explored the efficacy of pembrolizumab [51]. This Phase II study included three cohorts that received single-agent pembrolizumab. Cohort 1 included patients with Response Evaluation Criteria in Solid Tumors (RECIST)-measurable, PD-L1 positive disease, using a combined positive score (CPS) of ≥1 to define positivity (n = 133), where CPS is the number of PD-L1 positive cells, including immune cells and tumor cells, divided by the total number of tumor cells, multiplied by 100 [51,52]. Cohort 2 included patients with RECIST-measurable, PD-L1 negative disease (n = 65), and Cohort 3 included patients with bone-predominant disease, irrespective of PD-L1 status (n = 59). All patients also had to have been previously treated with one or more next-generation hormonal therapies and one or two lines of prior chemotherapies, one of which had to have been docetaxel. Pembrolizumab 200 mg IV was given once every 3 weeks for up to 35 cycles. Median treatment duration was brief; 2.1 months in Cohort 1, 1.6 months in Cohort 2, and 3.2 months in Cohort 3, with the most common reasons for treatment discontinuation being disease progression and adverse events.

ORR was 5% in Cohort 1, 3% in Cohort 2, and 5% in Cohorts 1 and 2 combined. In Cohort 1, 2 patients achieved a complete radiographic response, five patients had a PR, and six patients had stable disease for 6 months or longer. Within Cohort 2, two patients had a PR, and four patients had stable disease for 6 months or longer. Among the nine patients in Cohorts 1 and 2 with a radiographic response, four remained on treatment at the time of data cutoff, three experienced subsequent disease progression, and one died without disease progression [51]. This study shows that while responses to pembrolizumab in mCRPC may be few, responses can be durable. Although the study was not designed to detect a difference between PD-L1 positivity and negativity, outcomes were similar between the two.

Biomarker analysis was performed of patients in Keynote-199, evaluating tumor mutational burden (TMB), PD-L1 positive (combined positive score [CPS] ≥ 1) or negative (CPS < 1) disease, microsatellite instability (MSI), and tumor microenvironment-based 18-gene RNA expression profile; this abstract was presented at ASCO 2020 [53]. Higher TMB and higher PD-L1 CPS were associated with PSA response (*p* = 0.0015 and *p* = 0.046, respectively). However, small patient numbers did not allow authors to draw conclusions on biomarkers and ORR, disease control rate (DCR), or OS.

## 7. CTLA-4/PD-1 Combination

Interestingly, in a clinical trial where ipilimumab was given in the neoadjuvant setting with androgen-deprivation therapy (ADT), PD-1 and PD-L1 expression was significantly higher in patients who had been treated with ipilimumab plus ADT, compared to those treated with ADT alone, and compared to pretreatment biopsy specimens [54]. Anti-CTLA-4 therapy is thought to prime T cells, whereas anti-PD-1 therapy is involved later in activation of immune effector response at the cancer cell level [55]. Combination therapy with ipilimumab and nivolumab is being explored in several other tumor types, with observed improvements in OS in melanoma [56,57].

This inspired a clinical trial combining CTLA-4 blockade and PD-1 inhibition for advanced prostate cancer. Checkmate 650 is an ongoing Phase II clinical trial evaluating the combination of ipilimumab with nivolumab in mCRPC. The study is divided into two cohorts; Cohort 1 included 45 patients with mCRPC who had previously received second-generation hormone therapy, and Cohort 2 included 45 patients who had previously received second-generation hormone therapy as well as chemotherapy [58]. Patients received nivolumab 1 mg/kg and ipilimumab 3 mg/kg every 3 weeks for up to four cycles. Coprimary endpoints were ORR per RECIST and radiographic-PFS per the Prostate Cancer Clinical Trials Working Group 2 criteria [52,59].

ORR was 26% and 10% in Cohorts 1 and 2, respectively. In patients with a PD-L1 expression of 1% or greater, ORR was 50% in Cohort 1 and 25% in Cohort 2. Additionally, patients with a mutation in DNA damage repair, a homologous recombination deficiency, or an above-median TMB also had higher ORR. Importantly, Grade 3–4 treatment-related adverse events occurred in 39% of patients in Cohort 1, and 59% in Cohort 2. One Grade 5 event occurred in each cohort. 33.3%t and 35.6% discontinued treatments due to AEs in Cohorts 1 and 2, respectively, and only 33.3% and 24.4% of patients completed all four cycles of treatment [58].

While limited by significant toxicities, this study does lend support to the importance of identifying biomarkers, which could predict responses to immunotherapy. Checkmate 650 is ongoing and comparing ipilimumab plus nivolumab, versus ipilimumab alone, versus cabazitaxel (NCT02985957).

Another Phase II trial looked at the combination of ipilimumab + nivolumab specifically in patients with androgen-receptor splice variant 7 (AR-V7)-expressing circulating tumor cells. AR-V7-expressing metastatic prostate cancer results in constitutive activation of the androgen receptor and lacks the binding site for abiraterone and enzalutamide, rendering these tumors resistant to these therapies [60]. Biomarker analyses have shown significantly higher numbers of DNA-repair defects, which may make them more susceptible to immunotherapy [61].

Patients received 3 mg/kg of nivolumab + 1 mg/kg of ipilimumab every 3 weeks for four doses, followed by maintenance of 3 mg/kg of nivolumab every 2 weeks. Of 15 patients with AR-V7-expressing circulating tumor cells, two achieved a PSA response. ORR for patients with measurable soft-tissue disease was 25%. Six of these 15 patients were found to have DNA-repair deficiencies (DRD+). There was a trend seen towards DRD+ patients and responses, with 33% of DRD+ having a PSA response compared to 0% who were DRD− (*p* = 0.14), and ORR of 40% vs. 0%, respectively (*p* = 0.46). Larger prospective studies are needed to confirm the trends seen in this study [62].

## 8. PD-L1 Expression in Prostate Cancer

Many questions remain regarding the implications of PD-L1 positivity in prostate cancer. Ness et al. evaluated 535 patients who underwent prostatectomies and evaluated PD-1 expression on intratumoral lymphocytes, as well as PD-L1 expression in tumor epithelial cells. PD-1 positivity on intratumoral lymphocytes was scored as the number of positive stained cells per 0.6 mm diameter core, with 0 = 0–3 cells, 1 = 4–10 cells, 2 = 11–15 cells, and 3 = >15 cells. A score of ≥1.25 was considered high density. PD-L1 intensity was scored by immunohistochemistry staining on tumor epithelial cells, with 0 = no staining, 1 = weak staining, 2 = moderate staining, and 3 = strong staining. A high-intensity score was defined as ≥1 for tumor epithelial cells. They found that 39% of patients had PD-1 positive intratumoral lymphocytes; overall, these were sparse, with only 11% having a high density of PD-1 positive intratumoral cells. PD-L1 staining of tumor epithelial cells was positive in 92% of cases, with 59% having a high PD-L1 intensity score. They identified a trend towards a negative association between PD-L1 positive tumor epithelial cells and biochemical failure-free survival. In contrast, they found a trend towards worse clinical failure-free survival in patients with PD-1 positive lymphocytes. Specifically, in subgroups with features that portend unfavorable prostate cancer outcomes—age <65 years, pT3 stage, PSA > 10 ng/mL at diagnosis, and Gleason Grade 9—there was a significantly higher risk of clinical failure if these patients had a high density of PD-1 positive intratumoral lymphocytes [63].

## 9. Enzalutamide’s Potential Impact on PD-L1 Expression

Patients with prostate cancer who are progressing on enzalutamide have been shown to have higher levels of PD-L1 on dendritic cells and PD-1 positive T cells relative to patients who have not received enzalutamide or who are responding to it [64]. This creates interest in combining enzalutamide with immunotherapy to enhance response to PD-1 blockade potentially. Several trials have evaluated this combination with conflicting results.

A 2016 Phase II study evaluated this combination of immuno-hormonal therapy with enzalutamide and pembrolizumab in patients with mCRPC [65]. Patients were eligible if they had evidence of progressive disease on enzalutamide, either in the form of rising PSA or radiographic progression. Patients were continued on enzalutamide and were given pembrolizumab every 3 weeks for four doses total. Ultimately, 58 patients were evaluated.

Three of the first 10 patients experienced significant antitumor activity, starting with PSAs of 46, 71, and 2503 ng/mL, all declining to <0.1 ng/mL. Of these three patients, two had measurable soft tissue disease prior to treatment, and both experienced a PR to treatment, with one experiencing a response in liver metastases. All three remained progression-free at 30, 55, and 16 weeks of follow-up. Of the seven remaining patients, three had stable disease at 30, 47, and 50 weeks, while four had no evidence of clinical benefit. Of the three dramatic responders, two had a baseline tissue biopsy prior to treatment with pembrolizumab, and one was revealed to have microsatellite instability, a known feature that predicts response to immunotherapy [66]. In the initial cohort of 28 patients, five (18%) had a PSA decline to 0.2 ng/mL, and three of 12 patients with measurable disease had an objective response. In the next 30-patient cohort expansion, results were similar, with six (20%) with PSA response, and six of 27 (20%) with measurable disease had an objective response [67].

The Keynote 199 Phase II trial expanded to include two additional cohorts evaluating the role of combined immunohormonal therapy with pembrolizumab and enzalutamide. Cohort 4 included patients with mCRPC progressing on enzalutamide who had RECIST-measurable disease, and Cohort 5 included patients with mCRPC progressing on enzalutamide who had bone-predominant disease; neither group had yet received cytotoxic chemotherapy for castration-resistant disease. Patients were continued on enzalutamide and received pembrolizumab every 3 weeks for up to 35 cycles until intolerance or disease progression. In Cohort 4, there was a 12% confirmed radiographic response rate with two CRs and eight PRs; however, including unconfirmed responses, meaning patients who did not have adequate follow-up for confirmatory imaging or had poor image quality for repeat imaging, there was a 24% response rate. Responses were durable, with 60% having a continued response at 6 months [68]. Importantly, two patients in Cohort 4 died of immune-related adverse events.

PD-L1 inhibition with atezolizumab, in combination with enzalutamide, has also been explored. Outcomes from the Phase III trial IMbassador250 were presented at the American Association for Cancer Research 2020. This trial included 759 patients with mCRPC who had either progressed on abiraterone and docetaxel or were ineligible or refused taxane-based therapy. Patients were randomly assigned to receive enzalutamide 160 mg daily plus atezolizumab 1200 mg every 3 weeks or enzalutamide 160 mg daily alone. The primary endpoint was OS. After a median follow-up of 11 months, OS in the combination arm was 15.2 months versus 16.6 months in the enzalutamide alone arm. Treatment-related adverse events occurred in 77.8% of patients in the combination group and 51.1% of patients in the enzalutamide alone group, with Grade 3–4 events occurring in 28.3% and 9.6% of patients, respectively. The trial was terminated early due to lack of efficacy. In a subgroup analysis looking at PD-L1 expression, there was no difference in OS between patients with PD-L1 < 1%, PD-L1 ≥ 1%, or PD-L1 ≥ 5% [69].

Given the findings that patients who are no longer responding to enzalutamide may have higher levels of PD-L1 positive dendritic cells, there are several ongoing studies exploring the combination of enzalutamide with pembrolizumab in an effort to augment response to PD-1 inhibition. Phase III trials Keynote-991 and Keynote-641 are currently enrolling. Keynote-641 will evaluate the combination of enzalutamide and pembrolizumab vs. enzalutamide and placebo (NCT03834493), whereas Keynote-991 will explore this combination in patients who are hormone-sensitive (NCT04191096).

## 10. Future Directions

Studies are ongoing to understand better why some prostate cancer patients exhibit dramatic responses to immunotherapy, whereas the majority do not. There are six ongoing Phase III studies evaluating CPIs in metastatic prostate cancer in combination with chemotherapy, hormone therapy, targeted therapy, or with other CPIs (Table 1). These studies hope to clarify how to improve T cell activation and to decrease the immunosuppressive milieu in tumors.

## 11. Bi-Specific T-Cell Engagers

Bi-specific T-cell engagers (BiTEs) are another evolving area in prostate cancer treatment. BiTEs are bispecific monoclonal antibodies, one of which binds to T-cells through the CD3 receptor, the other of which is targeted against a tumor-specific marker. There are several potential benefits to this approach, including it is an “off-the-shelf” therapy that does not have to be manufactured for each patient, and also, the T cells do not have to have a T cell receptor specific to the tumor. Thus far, the only FDA-approved BiTE is blinatumomab, which targets CD19+ malignancies and is used in the treatment of B-cell acute lymphoblastic leukemia [70].

Pasotuxizumab is the first-in-human trial of a prostate-specific membrane antigen (PSMA)-targeting BiTE (NCT01723475). PSMA is a frequently highly expressed poorly differentiated and castrate-resistant prostate cancer and is infrequently expressed in other tissues, making it an attractive target for prostate cancer treatment [71,72]. The Phase I trial with pasotuxizumab showed dose-dependent PSA responses, with two long-term responses lasting 14 and 19.4 months [73].

HPN424 is another BiTE directed against PSMA. HPN424 is actually a tri-specific T cell-activating construct, which also has an albumin-binding domain, with the goal of extending the compound’s half-life in vivo. This drug is currently undergoing a Phase I, multicenter, open-label dose-escalation and dose-expansion study for patients who have received at least two prior treatment regimens for mCRPC and have evidence of disease progression on most recent systemic treatment (NCT03577028). Notable adverse events include cytokine release syndrome, which is consistent with what is seen with other BiTE therapies related to cytokine release from activated T cells [74].

AMG 160 is another half-life extended BiTE which is actively being explored in patients with mCRPC, both as monotherapy and in combination with pembrolizumab for patients who are refractory to novel hormonally therapy and who have received, or are unfit for or refused, taxane therapy. AMG 160 also uses PSMA as a target. Interestingly, AMG 160 has been shown to upregulate PD-1 in the tumor microenvironment. Preclinical studies have shown the killing of PSMA-positive human prostate tumor cells in vitro and tumor regression in vivo. The Phase I first-in-human study of AMG 160 is ongoing (NCT03792841) [75].

Another potential target which is under evaluation is six transmembrane epithelial antigens of the prostate 1 (STEAP1), which is highly expressed in prostate cancer. AMG 509 is a bispecific monoclonal antibody that uses an anti-STEAP1 domain in combination with an anti-CD3 domain to bind T cells. AMG 509 is currently being explored in a Phase I trial for relapsed/refractory mCRPC for patients who are refractory to novel hormonal therapy and have failed 1–2 taxane regimens, or not candidates for or refuse taxane therapy (NCT04221542) [76].

## 12. Chimeric Antigen Receptor T-Cell Therapy 

Another emerging field that has shown promise is chimeric antigen receptor T-cell (CAR-T) therapy. CAR-T therapy involves taking a patient’s T cells, engineering them to express a T cell receptor directed against a certain antigen, expanding the cells, and reinfusing them back into the patient. CAR-Ts directed against CD19 have been approved for the treatment of relapsed/refractory acute lymphoblastic leukemia and diffuse large B-cell lymphoma. CAR-Ts are also designed to express costimulatory domains, which may interact with ligands or receptors on antigen-presenting cells, tumor cells, or on the T cell itself [77].

While CAR-Ts have changed the treatment landscape for some hematologic malignancies, applying them to solid tumors, such as prostate cancer, has been challenging. CAR-Ts may be rendered less effective due to the inhibitory tumor microenvironment of prostate cancers. Additionally, changes in the tumor microenvironment related to aberrant angiogenesis mediated by vascular endothelial growth factor receptor (VEGF) may limit the ability of CAR-Ts to come into contact with solid tumors [78]. CAR-T designers have explored inserting chemokine receptor genes into CAR-Ts, which may enable more CAR-Ts to hone in on tumor cells. CAR-Ts expressing the chemokine receptor CXCR2, which is the receptor for tumor-derived IL-8, have been shown to migrate more efficiently towards IL-8 and to exhibit superior antitumor activity in vitro [79]. Additionally, attempts are being made to alter the immunosuppressive microenvironment through inhibition of transforming growth factor beta (TGFβ). TGFβ is secreted by some solid tumors and allows for immune system evasion; it has been shown to suppress T cell effector function and impact T-cell differentiation, driving T-cells into the regulatory phenotype [80]. TGFβ-specific CAR-T cells take advantage of this by using TGFβ as a stimulant to activate T-cells.

Two prostate-specific antigens are being explored in Phase I trials in prostate cancer thus far using PSMA and prostate stem cell antigen (PSCA), which is primarily expressed in prostate cancer cells and is expressed in advanced prostate cancer [81]. Two Phase I trials have published data. An anti-PSMA CAR-T cell was evaluated in five patients. Two of these patients had a PSA decline ≥50%, but T cell persistence appeared to be brief [82]. Another trial evaluated anti-PSMA CAR-T cells in seven patients. In the lower dose cohort, two patients had stable disease for 16 and 6 months, and the other two patients progressed. Of three patients in the higher dose cohort, all three developed fever up to 39 °C, consistent with what is seen in cytokine release syndrome. CAR-T cells were detected in circulation for up to 2 weeks [83]. Numerous Phase I trials exploring CAR-T cells in prostate cancer are ongoing.

## 13. Conclusions

Much remains to be understood regarding immunotherapy’s role and sequencing in metastatic prostate cancer. While positive results are not seen in the majority of patients, those that are observed can be dramatic, and patients can be in an apparent remission for many months or even years. Ongoing studies are attempting to elucidate the implications of PD-L1 expression, enzalutamide’s impact on PD-L1 expression, and the microbiome’s potential role in impacting response to immunotherapy. Beyond vaccine therapies and checkpoint blockade, new therapies, such as BiTEs and CAR-T cells, offer intriguing and promising avenues for the treatment of advanced prostate cancer.

## Figures and Tables

**Table 1:** Ongoing Phase III Clinical Trials Evaluating checkpoint inhibitors (CPIs) in Metastatic Prostate Cancer.

NCT	Title	Immunotherapy	Phase	Primary Endpoint	Status
NCT04191096	Efficacy and safety of pembrolizumab (MK-3475) plus enzalutamide plus androgen deprivation therapy (ADT) versus placebo plus enzalutamide plus ADT in participants with metastatic hormone-sensitive prostate cancer (mHSPC) (MK-3475-991/KEYNOTE-991)	Pembrolizumab	III	rPFS, OS	Recruiting
NCT03834493	A study of pembrolizumab (MK-3475) plus enzalutamide versus placebo plus enzalutamide in participants with metastatic castration-resistant prostate cancer (mCRPC) (MK-3475-641/KEYNOTE-641)	Pembrolizumab	III	OS, rPFS	Recruiting
NCT03834506	A study of pembrolizumab (MK-3475) plus docetaxel versus placebo plus docetaxel in chemotherapy-naïve metastatic castration-resistant prostate cancer (mCRPC) (MK-3475-921/KEYNOTE-921)	Pembrolizumab	III	OS, rPFS	Recruiting
NCT03834519	A study of pembrolizumab (MK-3475) plus olaparib versus abiraterone acetate or enzalutamide in metastatic castration-resistant prostate cancer (mCRPC) (MK-7339-010/KEYLYNK-010)	Pembrolizumab	III	OS, rPFS	Recruiting
NCT04100018	A study of nivolumab or placebo in combination with docetaxel in men with advanced castration-resistant prostate cancer	Nivolumab	III	rPFS, OS	Recruiting
NCT03879122	A trial of immunotherapy strategies in metastatic hormone-sensitive prostate cancer	Nivolumab, ipiliumumab, or nivolumab + ipilimumab	III	OS	Recruiting
